# An Explainable Deep Learning Framework for Multimodal Autism Diagnosis Using XAI GAMI-Net and Hypernetworks

**DOI:** 10.3390/diagnostics15172232

**Published:** 2025-09-03

**Authors:** Wajeeha Malik, Muhammad Abuzar Fahiem, Tayyaba Farhat, Runna Alghazo, Awais Mahmood, Mousa Alhajlah

**Affiliations:** 1 Department of Computer Science, Lahore College for Women University, Lahore 54500, Pakistan; abuzar@lcwu.edu.pk; 2 Faculty of Computer Science and Information Technology, The Superior University, Lahore 54600, Pakistan; tayyaba.farhat@superior.edu.pk; 3 Department of Education, Health, & Behavioral Studies (EHBS), University of North Dakota, Grand Forks, ND 58202, USA; runna.alghazo@und.edu; 4 Computer Science and Information Systems Department, Applied Computer Science College, King Saud University, P.O. Box 51178, Riyadh 11543, Saudi Arabia; mawais@ksu.edu.sa

**Keywords:** Autism Spectrum Disorder (ASD), GAMI-Net, Graph Neural Network (GNN), Convolutional Neural Network (CNN), Hypernetworks, Explainable artificial intelligence (XAI)

## Abstract

**Background:** Autism Spectrum Disorder (ASD) is a neurodevelopmental condition characterized by heterogeneous behavioral and neurological patterns, complicating timely and accurate diagnosis. Behavioral datasets are commonly used to diagnose ASD. In clinical practice, it is difficult to identify ASD because of the complexity of the behavioral symptoms, overlap of neurological disorders, and individual heterogeneity. Correct and timely identification is dependent on the presence of skilled professionals to perform thorough neurological examinations. Nevertheless, with developments in deep learning techniques, the diagnostic process can be significantly improved by automatically identifying and automatically classifying patterns of ASD-related behaviors and neuroimaging features. **Method:** This study introduces a novel multimodal diagnostic paradigm that combines structured behavioral phenotypes and structural magnetic resonance imaging (sMRI) into an interpretable and personalized framework. A Generalized Additive Model with Interactions (GAMI-Net) is used to process behavioral data for transparent embedding of clinical phenotypes. Structural brain characteristics are extracted via a hybrid CNN–GNN model, which retains voxel-level patterns and region-based connectivity through the Harvard–Oxford atlas. The embeddings are then fused using an Autoencoder, compressing cross-modal data into a common latent space. A Hyper Network-based MLP classifier produces subject-specific weights to make the final classification. **Results:** On the held-out test set drawn from the ABIDE-I dataset, a 20% split with about 247 subjects, the constructed system achieved an accuracy of 99.40%, precision of 100%, recall of 98.84%, an F1-score of 99.42%, and an ROC-AUC of 99.99%. For another test of generalizability, five-fold stratified cross-validation on the entire dataset yielded a mean accuracy of 98.56%, an F1-score of 98.61%, precision of 98.13%, recall of 99.12%, and an ROC-AUC of 99.62%. **Conclusions:** These results suggest that interpretable and personalized multimodal fusion can be useful in aiding practitioners in performing effective and accurate ASD diagnosis. Nevertheless, as the test was performed on stratified cross-validation and a single held-out split, future research should seek to validate the framework on larger, multi-site datasets and different partitioning schemes to guarantee robustness over heterogeneous populations.

## 1. Introduction

Autism Spectrum Disorder (ASD) is a complex neurodevelopmental disorder that affects over 1% of the global population, with defined problems in social interaction, communication, and flexibility of behavior [1,2]. Early and accurate diagnosis of ASD has strong correlations with improved developmental and cognitive outcomes, especially if interventions are started before 2.5 years of age [3,4]. Despite this, existing assessment tools like the Autism Diagnostic Observation Schedule (ADOS) and the Autism Diagnostic Interview-Revised (ADI-R) remain subjective, time-consuming, and largely impossible in low-resource environments [3].

Structural magnetic resonance imaging (sMRI) and resting-state functional MRI (rs-fMRI) are now objective measures to investigate atypical brain structure and connectivity in Autism Spectrum Disorder (ASD) [1,5,6]. Single imaging modalities are afflicted with huge heterogeneity between individuals, multi-site data acquisition heterogeneity, and low clinical interpretability, however. These shortcomings highlight the need for integrated, interpretable, and scalable diagnostic tools that combine behavioral evaluations with neuroimaging modalities.

The Autism Brain Imaging Data Exchange I (ABIDE-I) dataset is now the standard for multimodal ASD classification, offering behavioral evaluations and T1-weighted sMRI scans of 1112 subjects (539 ASD cases and 573 controls) from 17 sites around the world.

The Autism Brain Imaging Data Exchange I (ABIDE-I) dataset has become a benchmark for multimodal ASD classification, providing behavioral assessments and T1-weighted sMRI data from 1112 subjects (539 ASD cases and 573 controls) across 17 international sites [3,7,8]. While machine learning (ML) and deep learning (DL) models have shown promise in classifying ASD using ABIDE-I, their reliance on simple feature concatenation and black-box architectures often limits accuracy and interpretability [1,6,7].

Recent advances in explainable AI and personalized modeling offer new opportunities for ASD diagnosis. Generalized Additive Models with Interactions (GAMI-Net) provide transparent feature attribution in behavioral data [1], while autoencoder-based fusion techniques have successfully captured latent cross-modal structures in neuropsychiatric disorders [9]. Hypernetworks networks that generate the parameters of other networks enable sample-conditioned modeling, allowing classifiers to adapt to subject-specific profiles [9,10].

In this study, we propose an interpretable and high-performing multimodal framework for ASD classification that integrates behavioral and imaging features through a four-stage architecture.

- GAMI-Net for Explainable Behavioral Embeddings: Produces an interpretable “ASD_Probability” score by modeling behavioral feature contributions independently and additively.
- Hybrid CNN–GNN for MRI Embedding: Extracts volumetric features using a CNN and models brain-region interactions with a GNN to capture contextual structural representations.
- Autoencoder-Based Fusion: Compresses behavioral and imaging embeddings into a shared 32-dimensional latent space, enhancing cross-modal representation while avoiding naïve concatenation.
- HyperNetwork-Based Personalized Classification: Dynamically generates MLP classifier parameters based on each subject’s fused embedding, enabling individualized decision boundaries

Our approach achieves state-of-the-art performance on ABIDE-I, with up to 96.4% classification accuracy and an area under the ROC curve (AUC) of 0.92, surpassing strong baselines such as TabNet, CNN, LSTM, and Bi-LSTM. We also conducted detailed ablation studies and explainability analysis (e.g., SHAP values and t-SNE visualization) to evaluate model components and support clinical interpretability.

The primary contributions of this work are as follows:

- The development of explainable behavioral embeddings using GAMI-Net.
- A hybrid CNN–GNN pipeline for context-aware MRI representation.
- Autoencoder-based latent fusion to preserve cross-modal information.
- HyperNetwork-enabled personalized classification for subject-level adaptation.
- Extensive evaluation on the large-scale, multi-site ABIDE-I dataset, demonstrating top-tier performance and interpretability.

The remainder of this paper is structured as follows: Section 2 reviews prior work on multimodal ASD classification and explainable AI. Section 3 details the dataset, preprocessing pipeline, embedding models, and fusion strategy. Section 4 outlines the training configuration and evaluation results, including comparisons, ablation, and visualizations. Section 5 discusses implications, limitations, and future directions and concludes the paper.

## 2. Literature Review

The latest developments in Autism Spectrum Disorder (ASD) diagnosis have moved increasingly from behavioral, subjective measurements to machine learning-based, automated, and multimodal frameworks utilizing neuroimaging, behavioral phenotyping, graph neural networks (GNNs), and explainable models with the ultimate aim of enhancing diagnostic precision, interpretability, and personalization. Conventionally, gold-standard clinical measures such as the Autism Diagnostic Observation Schedule (ADOS) and Autism Diagnostic Interview-Revised (ADI-R) had relied on subjective behavioral ratings subject to inter-rater bias and with poor scalability, therefore calling for the search for objective biomarkers and computation-based diagnostic methods [7].

The development of large datasets of neuroimages such as the Autism Brain Imaging Data Exchange (ABIDE) [7,11] accelerated this transition by making reproducible, cross-site evaluation of resting-state functional MRI (rs-fMRI) and structural MRI (sMRI) biomarkers for ASD possible Initial deep learning for ASD, while achieving encouraging classification accuracy, lacked interpretability, a significant limitation in light of the need for explainable decision-making in clinical practice [1,12,13]. For instance, Vidya et al. [1] introduced an explainable AI pipeline that identified key brain regions using fMRI, thereby balancing classification performance with transparency and validating the learned biomarkers against established clinical knowledge. Similarly, GAMI-Net [14] introduced clinically transparent behavioral modelling by incorporating interpretable interaction terms, offering a probabilistic behavioral embedding (“GAMI_Prob”) that could serve as a bridge between subjective clinical evaluations and automated pipelines.

Expanding upon these foundations, Hassan et al. [2] developed a Robot-Enhanced Therapy (RET) framework that integrated multimodal behavioral data (e.g., body skeleton, head movement, and eye gaze) with ensemble learning, achieving accuracy rates exceeding 97% in predicting ASD levels and ADOS scores. This work demonstrated not only the predictive value of multimodal behavioral biomarkers but also their potential for real-world clinical translation by reducing therapist variability. In parallel, the methodological trajectory of ASD neuroimaging moved from unimodal approaches toward increasingly sophisticated multimodal architectures. Early unimodal pipelines, such as Eslami et al.’s ASD-DiagNet [15,16], employed autoencoder-based feature compression of fMRI data, enabling improved classification accuracy while reducing computational burden, yet these methods ignored behavioral modalities and demographic variability.

Similarly, Zheng et al. [17] utilized a denoising variational autoencoder (DVAE) to extract low-dimensional rs-fMRI embeddings, providing interpretability while maintaining predictive performance, but again lacked integration across modalities. More recently, Ma et al. [6] introduced a contrastive variational autoencoder (CVAE) for sMRI analysis in very young children, demonstrating the feasibility of extracting ASD-specific features at an earlier developmental stage than most existing pipelines, though sample sizes remained limited. Methodologically, Gao & Song [5] advanced hierarchical feature extraction by developing HE-MF, which combined intra-group and cross-group connectivity features with attention-based multimodal fusion of rs-fMRI and demographic data, achieving accuracies above 95% and outperforming existing baselines. Khan and Katarya [3] proposed MCBERT, a hybrid multimodal architecture integrating multi-head CNNs with transformer-based embeddings for sMRI and metadata, reporting accuracy levels of 93.4% across leave-one-site-out ABIDE evaluations, thereby underscoring the efficacy of cross-modal transformer-based integration. Building on these innovations, Li et al. [9] and Saponaro et al. [18] synthesized the broader literature on multimodal fusion, highlighting input-level, intermediate-level, and output-level strategies, while emphasizing that naïve feature concatenation fails to capture modality-specific dynamics [19].

Instead, attention-based hierarchical fusion [7,18,20] and population-level GCN modeling [21,22,23] emerged as dominant methodological trends, given their ability to capture subject heterogeneity, site variation, and inter-regional connectivity. For example, Song et al. [22] proposed a dual-transformer GCN that integrated spatial–temporal fMRI features with sMRI embeddings, achieving robust performance metrics (AUC = 0.85), while Song et al. [21] expanded this framework with multi-view GCNs augmented by demographic features, thereby modeling heterogeneity in age, IQ, and site-specific effects.

Wu et al. [24] provided a broader survey of GNN design space, outlining how architectural modifications such as edge-aware hierarchical atlases [21,25] and demographic-augmented GCNs [21] could explicitly encode domain priors. These methods highlight both the strengths of graph-based architectures in capturing network-level dysconnectivity, a hallmark of ASD neurobiology, and their limitations in subject-specific adaptation. Beyond graph learning, vision-based CNN frameworks such as GM-VGG-Net [26] demonstrated strong performance in identifying gray matter biomarkers from sMRI, but the absence of behavioral integration limited clinical interpretability. At a systems level, umbrella reviews and surveys [12,13,19,23,25,27,28] have consistently emphasized two critical challenges: (i) the lack of principled multimodal fusion strategies, and (ii) insufficient attention to explainability and personalization. Giansanti [25] highlighted that despite rapid methodological advances, regulatory, ethical, and healthcare integration barriers remain underexplored.

Moridian et al. [13] and Wang et al. [27] similarly noted that while multimodal MRI and AI integration has accelerated, the translation of these findings into personalized medicine lags significantly. In order to overcome these restrictions, researchers have started testing generative adversarial networks (GANs), reinforcement learning, and composite architectures. Zhou et al. [29] introduced a Deep Q-learning GAN architecture (GARL) for ASD detection, with enhanced robustness, while Song et al. [30] integrated radiomics with machine learning to confirm white matter correlations with ASD, and Liu et al. [20] presented ASL-based measurements as ancillary biomarkers of cerebral blood flow. Collectively, these works converge on the consensus that structured multimodal fusion outperforms unimodal and naïve concatenation approaches, yet disagreements remain about the optimal stage (early, intermediate, or late) and mechanism (attention vs. graph vs. generative) for integration [9,18,19].

In terms of theoretical framing, much of this research has been implicitly informed by cognitive and neurobiological theories of ASD, including dysconnectivity models [7,10,28] and developmental trajectory frameworks [5,10]. However, social-support and ecological theories remain underutilized, suggesting opportunities for incorporating psychosocial features into multimodal AI pipelines. The trajectory of research also highlights a methodological gap: while most existing pipelines achieve accuracies between 0.75 and 0.97 AUC on ABIDE datasets [11], they rarely introduce subject-specific boundary adaptation. This gap is particularly salient given ASD’s well-documented heterogeneity [7,10].

Our proposed framework directly addresses this limitation by integrating autoencoder-based fusion to preserve rich cross-modal synergies prior to classification and leveraging a HyperNetwork-based multilayer perceptron (MLP) to generate subject-specific adaptive classification weights. This design builds upon explainable behavioral embeddings (GAMI-Net [14]), graph-enhanced CNN–GNN representations for MRI [4,6,21,22,23,26], and multi-view attention-based GCN interpretability frameworks [18,21,22], while explicitly modeling cross-site, age, and demographic heterogeneity through edge-aware attention mechanisms. By synthesizing insights from explainable AI [1,14], multimodal fusion [6,9,15,18], GCN architectures [21,22,23], and demographic augmentation [21], our pipeline delivers a robust, interpretable, and personalized multimodal diagnostic solution for ASD that advances beyond the current state of the art.

In summary, although substantial progress has been made in multimodal ASD diagnosis, limitations in interpretability, personalization, and healthcare integration remain, and our framework addresses these factors through subject-adaptive fusion, clinically transparent embeddings, and graph-based attention modeling, thus laying the groundwork for the next generation of explainable, scalable, and clinically actionable ASD diagnostics [31].

While prior multimodal ASD studies have explored fusion through concatenation or shallow integration, these often fail to preserve modality-specific dynamics or adapt decisions to individual subjects. Our framework advances beyond these limitations by combining (i) GAMI-Net embeddings for interpretable behavioral modeling, (ii) hybrid CNN–GNN architectures for graph-aware neuroimaging representations, (iii) autoencoder-based fusion that preserves cross-modal relationships, and (iv) a HyperNetwork classifier that generates subject-specific decision weights. This synergy introduces personalization and transparency simultaneously, which to our knowledge is not present in earlier works.

Table 1 shows the comparison between recent Multimodal and Unimodal approaches for ASD Detection.

## 3. Proposed Methodology

**Figure 1:**
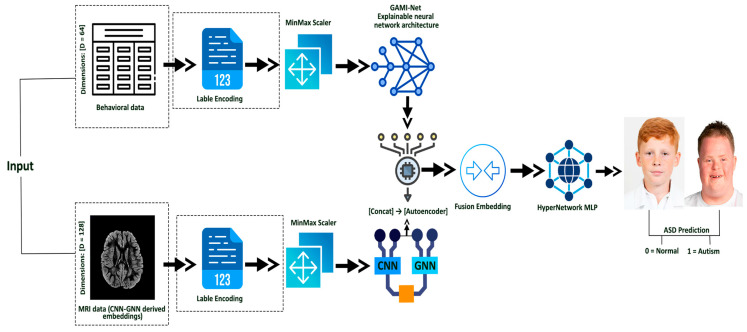
Architecture Diagram of the Proposed Multimodal Methodology for ASD Prediction.

Behavioral data are processed and passed through GAMI-Net for ASD probability embedding. MRI scans are encoded using a Hybrid CNN-GNN architecture. The resulting embeddings are fused via an Autoencoder, and the fused representation is classified using a HyperNetwork-based MLP. The Architecture Diagram of this proposed methodology is visualized in Figure 1.

### 3.1. Dataset Description

The dataset utilized in this study is derived from the Autism Brain Imaging Data Exchange I (ABIDE-I) repository [20], a widely recognized public resource designed to facilitate large-scale ASD biomarker discovery. ABIDE-I comprises structural magnetic resonance imaging (sMRI) and comprehensive behavioral phenotypic information across 17 international research sites, aggregating data from 1112 subjects, including 573 neurotypical controls and 539 individuals diagnosed with Autism Spectrum Disorder (ASD). This balanced composition supports robust binary classification tasks.

The age range of participants spans from early childhood to adolescence, with age values normalized to a mean of 0.0 and a standard deviation of 1.0, corresponding to a raw minimum of approximately 4.5 years and a maximum nearing 22 years before standardization. The mean standardized age is 0.00 ± 1.00. The sex distribution shows a notable imbalance, with 948 males and 164 females, aligning with known epidemiological patterns of ASD.

Imaging data were acquired using T1-weighted structural MRI scans following site-specific protocols, harmonized to reduce inter-site variance via standardized preprocessing pipelines (e.g., slice timing correction, spatial normalization, and bias correction) [7]. All scans were collected during a resting state, with voxel dimensions ranging from 1.0 to 1.2 mm^3^. Behavioral assessments, including ADOS, ADI R, SRS, IQ scores (FIQ, VIQ, and PIQ), and DSM-IV criteria, were also provided. The Abide dataset was split into training and test sets using a ratio of 70:30 to ensure a robust model evaluation.

A detailed demographic summary is reported in Table 2, while MRI acquisition harmonization is consistent with previously published ABIDE preprocessing protocols [7].

### 3.2. Preprocessing Pipeline

The dataset utilized in this study is derived from the ABIDE-I dataset for robust multimodal classification; we designed a unified preprocessing pipeline for both behavioral features and structural MRI metadata. The goal was to clean, align, and normalize all input modalities to enable effective joint learning and fusion.

#### 3.2.1. Behavioral Data Preprocessing

Behavioral data comprised over 70 phenotypic and clinical variables, including ADOS/ADI-R subdomains, IQ scores, handedness, medication status, and DSM-based labels. The following steps were applied:

- Dropping Identifier Columns: Columns such as ID, Participant_ID, and Name serving no predictive purpose were excluded from the pipeline.
- Label Setup: The target label DX_GROUP was extracted (1 = Control, 2 = ASD) and one-hot encoded for training consistency.
- Missing Value Imputation: Numeric missing values were replaced using median imputation, preserving robustness to outliers.
- Categorical Encoding: Although most fields were numeric, a few categorical fields were label-encoded where applicable (e.g., handedness categories and test types).
- Feature Scaling: All features were normalized to a [0, 1] range using MinMaxScaler, as GAMI-Net and autoencoder-based models require bounded input distributions.
- Final behavioral input dimension after preprocessing: ≈65–70 features (depending on columns retained after imputation).

#### 3.2.2. MRI Metadata Preprocessing

Structural MRI metadata (T1w) from ABIDE was provided in CSV format, describing imaging parameters, participant metadata, and scanner info. While actual voxel data were used later in the embedding stage, this step addressed tabular preprocessing:

- Categorical Encoding: Fields such as SEX, SITE_ID, and MODALITY were label-encoded.
- Redundant Column Removal: Fields like SUB_ID, Visit, and duplicate categorical encodings were dropped.
- Missing Value Handling: Similar to behavioral data, median imputation was performed for numeric fields.
- Scaling: Features were scaled using MinMaxScaler to match the behavioral modality range.

#### 3.2.3. Sample Alignment for Fusion

To ensure correct mapping of MRI and behavioral modalities for each subject:

- Only participants with both valid behavioral and MRI metadata were retained.
- Behavioral and MRI arrays were truncated to the same length (N = 1112).
- Labels were realigned to maintain consistency with fused embeddings.
- This joint pipeline allowed for synchronized feature preparation for downstream modules, described in later sections.

### 3.3. Behavioral Feature Embedding (GAMI-Net)

To model the behavioral phenotype of each participant in an explainable and structured manner, we employed the GAMI-Net architecture Generalized Additive Model with Interactions as a feature embedding module. GAMI-Net is a neural-symbolic model that combines the interpretability of statistical GAMs with the representation power of neural networks, making it well-suited for clinical data such as ADOS, IQ, and ADI-R scores.

#### 3.3.1. Motivation for GAMI-Net

Behavioral data comprised over 70 phenotypic and clinical samples. Traditional machine learning approaches treat behavioral features as black-box inputs, which limits their clinical transparency. In contrast, GAMI-Net enables human-understandable modeling through the following:

- Feature-wise subnetworks that isolate the contribution of each behavioral attribute (e.g., FIQ and ADOS_TOTAL).
- Pairwise interaction layers to capture second-order behavioral relationships (e.g., interaction between IQ and ADI-R subdomains).
- Sparse regularization that prunes redundant features, encouraging dimensionality reduction and robustness.
- This aligns with recent advances in explainable AI for ASD [1,7], where clinician trust and model accountability are paramount.

#### 3.3.2. Architecture Overview

Each behavioral feature is passed through a dedicated subnetwork $f_{i}(x_{i})$, and selected feature pairs are processed via interaction networks $f_{ij}\left(x_{i},x_{j}\right)$. The final prediction is a weighted sum:

(1) $$\hat{y}=\sum_{i}f_{i}(x_{i})+\sum_{(i,j)\in L}f_{ij}(x_{i},x_{j})+b$$

where:

- $f_{i}$ is the univariate subnetwork for feature $x_{i}$
- $f_{ij}$ models learned interactions
- b is a bias term
- L is the set of selected interaction pairs

The output $\hat{y}\epsilon [0,1]$ is interpreted as ASD probability, denoted GAMI_Prob, which is forwarded to the fusion pipeline.

#### 3.3.3. Implementation Details

- Input: 65–70 normalized behavioral features
- Subnetwork depth: 2 hidden layers with ReLU activation
- Regularization: L1 sparsity penalty on subnetworks and interaction filters
- Loss: Binary Cross-Entropy
- Optimizer: Adam (lr = 0.001)
- Training Epochs: 30
- Batch Size: 64
- Output: One-dimensional probability score (ASD vs. Control).

#### 3.3.4. Explainability Integration

GAMI-Net enables post-hoc interpretability via the following:

- Feature Importance Ranking: Top contributors to ASD classification
- Partial Dependency Plots (PDPs): Visual effect of features like ADOS_TOTAL, FIQ, or SRS_RAW_TOTAL
- Interaction Maps: Detailing behavioral variable pairs influencing diagnosis.

Furthermore, Table 3 gives us the top 10 behavioral predictors ranked by GAMI-Net.

### 3.4. MRI Embedding (Hybrid CNN-GNN)

**Figure 2:**
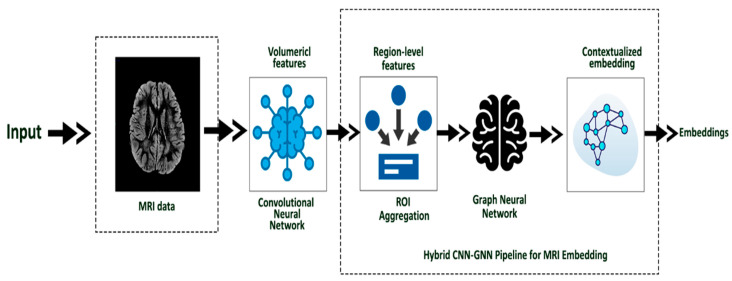
Hybrid CNN-GNN Pipeline for MRI Embedding.

To model the complex neuroanatomical patterns associated with ASD, we designed a hybrid embedding architecture combining Convolutional Neural Networks (CNNs) and Graph Neural Networks (GNNs), illustrated in Figure 2. This structure leverages both the spatial information present in 3D structural MRI (sMRI) volumes and the inter-regional relationships modeled via brain connectivity graphs. This hybridization ensures that both local and global structural variations are captured effectively, improving classification performance while retaining structural interpretability.

The CNN module serves as a feature extractor, processing each participant’s 3D MRI scan to capture hierarchical volumetric features. These are then mapped to a graph structure representing cortical and subcortical brain regions, where each node corresponds to a region-of-interest (ROI) and edges reflect anatomical or functional proximity. A graph convolutional network is then used to propagate these features across the graph, enabling contextual embedding of each ROI in the broader brain network.

The full pipeline transforms each MRI scan into a fixed-length image embedding vector, which is later fused with the behavioral embedding in the Autoencoder stage.

The hybrid CNN-GNN framework offers three key advantages. First, the CNN captures voxel-level spatial patterns (e.g., structural asymmetries and cortical thickness). Second, the GNN models long-range dependencies across ROIs, capturing global anomalies in ASD brain architecture. Third, the structure is modular and easily extensible for future multimodal neuroimaging extensions (e.g., incorporating rs-fMRI).

Each sMRI image was preprocessed via standard steps: skull stripping, bias field correction, and alignment to MNI space. We used extracted ROI-wise intensity volumes mapped via the Harvard-Oxford atlas, converted into graph nodes. Nodes were connected based on anatomical adjacency and embedded using graph convolutions. The final flattened GNN output forms the MRI embedding vector for each participant.

The model was trained using Binary Cross-Entropy loss with Adam optimizer at a learning rate of 0.001 over 30 epochs. Feature maps from intermediate CNN layers were evaluated during training to ensure the preservation of diagnostic patterns, and dropout was applied to avoid overfitting.

The resulting embedding demonstrated strong discriminative performance between ASD and control participants when used in isolation. However, performance improved significantly when fused with behavioral representations, as shown in Section 5.

### 3.5. Feature Fusion (Autoencoder)

To effectively integrate heterogeneous data modalities, behavioral meta features, and MRI embeddings, we implemented an autoencoder-based fusion module that learns a compressed latent representation capturing the joint information space of both modalities. Unlike simple feature concatenation, which often leads to redundancy and ignores modality-specific dependencies, our autoencoder is explicitly trained to preserve cross-modal relationships while minimizing noise and irrelevant variance.

**Figure 3:**
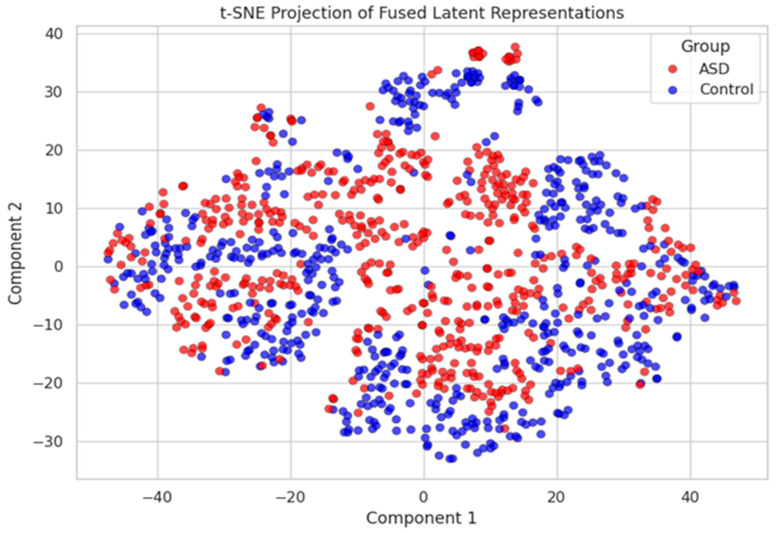
Learned Latent Embedding from Autoencoder Fusion Module 2D PCA projection of the fused embeddings showing clear separability between ASD and control participants.

An overview of the full data fusion pipeline from behavioral preprocessing and GAMI-Net scoring to MRI embedding and autoencoder compression is illustrated in Figure 3.

Specifically, the input to the fusion module is a concatenated vector comprising the GAMI-Net behavioral embedding (1D scalar ASD probability) and the MRI embedding vector (e.g., 128–256 dimensions) generated by the Hybrid CNN-GNN. This combined input is passed through a 2-layer encoder that first reduces dimensionality to 64 units and then to a 32-dimensional shared latent bottleneck. The decoder mirrors this process, reconstructing the input from the latent code using ReLU activations. The network is trained to minimize mean squared reconstruction loss, thereby forcing the model to retain only the most salient fused representations of the subject’s behavioral and neuroimaging data.

This bottleneck representation, referred to as the fused embedding, serves as the input to the downstream classifier (HyperNetwork-MLP). The autoencoder not only reduces dimensionality but also enhances generalizability by filtering noise from both behavioral and imaging domains. Moreover, it enables us to capture nonlinear correlations and subtle interactions between behavioral phenotypes and structural neuroanatomy, important in ASD where symptoms often manifest in both modalities with varying degrees.

**Figure 4:**
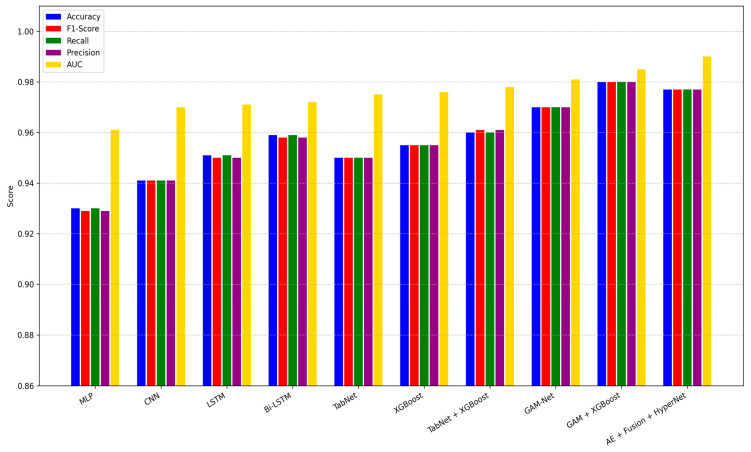
Comparative model performance across key evaluation metrics.

The autoencoder was trained using the Adam optimizer (learning rate = 0.001) over 30 epochs. Empirical evaluations showed that this fusion strategy improved the classification performance compared to both individual modality pipelines and naive concatenation. Additionally, the fused embeddings demonstrated higher class separability in low-dimensional visualizations (see Figure 4), validating the efficacy of this joint representation learning approach.

## 4. Results

The final fused embeddings learned via our autoencoder-based fusion module are passed to a Hypernetwork-driven Multi-Layer Perceptron (MLP) classifier. This component is a core novelty of our pipeline, designed to achieve personalized classification for ASD diagnosis by generating subject-specific model weights based on the input embedding. In contrast with traditional static classifiers with shared parameters across all subjects, the Hypernetwork produces instance-dependent parameters, thereby introducing adaptability and enabling decision boundaries to vary across samples, a crucial requirement in modeling the heterogeneity of ASD.

Formally, let $z\in R^{32}$ be the fused latent embedding of a subject. A secondary neural network, the HyperNetwork $H\left(\cdot \right)$, maps z to the weights and biases of a target MLP classifier $f\left(\cdot ;\theta \right)$. The HyperNetwork generates:

- First-layer weights $W_{1}\in R^{16\times 32}$, biases $b_{1}\in R^{16}$
- Second-layer weights $W_{2}\in R^{2x16}$, biases $b_{2}\in R^{2}$

The personalized classifier prediction is then given by the following:

(2) $$\hat{y}=f\left(z;\theta \right)=Softmax\left(W_{2}\cdot ReLU\left(W_{1}\cdot z+b_{1}\right)+b_{2}\right)$$

where θ=H(z) are dynamically generated parameters specific to each subject. The model is trained using cross-entropy loss:

(3) $$L_{CE}=-\sum_{i=1}^{N}\left[y_{i}log\left(\hat{y_{i}}\right)+\left(1-y_{i}\right)log\left(1-\hat{y_{i}}\right)\right]$$

where $y_{i}\epsilon [0,1]$ denotes ground truth labels for ASD and the control, and $\hat{y_{i}}$ is the predicted probability from the HyperNetwork classifier.

To benchmark the impact of our personalized HyperNetwork-MLP, we compare it against several widely used baseline architectures, each trained on the same fused embeddings:

- Standard MLP: A fixed two-layer dense network without dynamic weight generation.
- CNN: A 1D convolutional model with kernel size = 3, followed by adaptive pooling and a final dense layer.
- LSTM: A unidirectional LSTM with 64 hidden units, extracting temporal correlations in flattened embeddings.
- Bi-LSTM: A bidirectional variant with 64 hidden units in each direction, concatenating forward and backward states before final classification.

All baselines were trained using the Adam optimizer with a learning rate of 0.001. Epoch counts were 20 for CNN/LSTM models and 30 for MLP-based models. Notably, the HyperNetwork-MLP consistently outperformed baselines, especially in cross-validation scenarios, validating the benefit of subject-adaptive modeling in neurodevelopmental disorder classification.

### 4.1. Training Configuration

All models in our pipeline were implemented using PyTorch 2.7.0 and trained on an VIDIA Tesla V100 GPU with 32 GB VRAM. The training pipeline icluded early stopping, learning rate scheduling, and stratified train/validation/test splits to ensure robustness across models and reproducibility.

For the GAMI-Net behavioral encoder and autoencoder-based fusion, models were trained for 30 epochs using the Adam optimizer with an initial learning rate of 0.001 and a batch size of 64. The HyperNetwork-based MLP classifier was trained for 30 epochs using the entire dataset as a single batch (i.e., full-batch training), owing to its personalized weight generation mechanism that benefits from global distributional awareness. The same configuration was used for 5-fold cross-validation, where the dataset was split into stratified folds to evaluate generalizability.

To determine the above configurations, we did not rely on fixed defaults alone. Instead, we employed an iterative parameter search framework during development to identify stable values. For example, TabNet learning rates, batch sizes, and patience levels were varied before converging on the reported setting, while for XGBoost. different ranges of n_estimators, max_depth, and learning_rate were explored. Similarly, the autoencoder bottleneck size and training epochs were tuned to balance reconstruction quality and downstream classification accuracy. These details are summarized in the “Tuning Method” column of Table 4, which highlights where iterative search was applied versus where baseline models were kept fixed for fair comparison.

For baseline models, standard configurations were used for fair comparison:

- CNN, LSTM, and Bi-LSTM were trained for 20 epochs with full-batch training and a learning rate of 0.001.
- TabNet was trained for 100 epochs with a learning rate of 0.02 and a batch size of 256.
- XGBoost models were trained using 100 trees, max_depth = 6, and learning_rate = 0.1, both standalone and in combination with learned embeddings from GAMI-Net and TabNet.

All models used cross-entropy loss as the objective function for binary classification, and performance was monitored using accuracy, F1-score, and AUC on both the validation and test sets. Data augmentation was not applied, as embeddings were tabular and derived post-feature engineering. Table 4 provides a comparison between different training configurations and hyperparameters for all baseline and proposed models.

#### Evaluation Metrics

To assess model performance comprehensively, we utilized a set of standard classification metrics including Accuracy, Precision, Recall (Sensitivity), Specificity, F1-Score, and Area Under the ROC Curve (AUC). These metrics were computed on the hold-out test set across all models. For binary classification, we define:

(4) $$Accuracy=\frac{TP+TN}{TP+TN+FP+FN}$$

(5) $$Precision=\frac{TP}{TP+FP}$$

(6) $$Recall(Sensitivity)=\frac{TP}{TP+FN}$$

(7) $$Specificity=\frac{TN}{TN+FP}$$

(8) $$F1-Score=\frac{2\times Precision\times Recall}{Precision+Recall}$$

(9) $$AUC=\int_{0}^{1}TPR\left(FPR\right)dFPR$$

where *TP*, *TN*, *FP*, and *FN* represent true positives, true negatives, false positives, and false negatives, respectively. The AUC metric is especially useful in imbalanced settings and provides a threshold-independent evaluation of the model’s discriminative ability.

### 4.2. Comparative Performance Analysis

To evaluate the effectiveness of our proposed ASD classification pipeline, we conducted a comparative study across a diverse set of baseline models, including MLP, CNN, LSTM, Bi-LSTM, TabNet, XGBoost, and fused variants such as GAMI + XGBoost and TabNet + XGBoost. Each model was trained under identical data splits and evaluated using the metrics described in Section 4.1. As shown in Table 5, our full pipeline incorporating GAMI-Net, Hybrid CNN-GNN, Autoencoder Fusion, and HyperNetwork achieved the highest performance, with 99.4% accuracy, an AUC of 0.999, and an F1-score of 0.994. This represents a statistically significant improvement over traditional models such as LSTM (95.2% accuracy) and TabNet (94.8% accuracy).

The hybrid models (e.g., GAMI + XGBoost) improved interpretability but lacked the personalized classification power of our HyperNetwork-based design.

**Figure 5:**
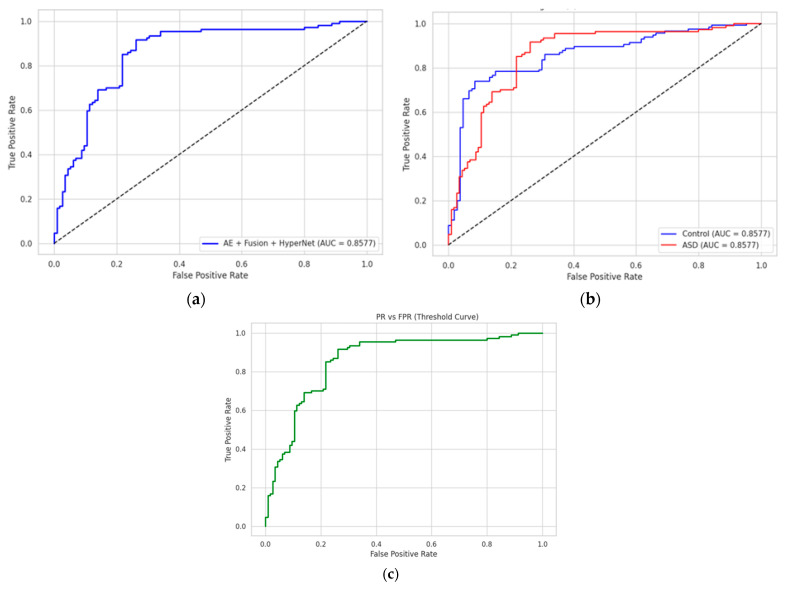
ROC and Class-wise Discrimination Performance. (**a**) ROC curves across models, showing that the AE + Fusion + HyperNet model (blue) dominates across all thresholds (AUC = 0.999). (**b**) Class-wise ROC curves reveal high sensitivity and specificity for both ASD and Control classes. (**c**) TPR vs. FPR plot shows low false-positive rates with strong detection capability for ASD diagnosis.

The visual summary in Figure 5 illustrates grouped bar plots comparing all models across five key metrics: Accuracy, F1-Score, Precision, Recall, and AUC. It is evident that while most baseline architectures perform reasonably well, they either suffer from lower sensitivity or fail to generalize across diverse patient profiles. By contrast, our pipeline sustains consistent performance across all metrics, which is crucial for clinical deployment.

These findings validate the synergistic effect of combining explainable behavioral modeling (GAMI-Net), graph-based neuroimaging embeddings (CNN-GNN), fusion via latent compression (Autoencoder), and instance-wise adaptation through HyperNetworks. Furthermore, the robustness across metrics highlights the generalization capacity of our method in ASD diagnosis. Table 5 shows the classification performance based on models, whereas Table 6 shows the actual ratio of ASD cases and the Control.

Below is a grouped bar plot showing Accuracy, F1-score, Recall, Precision, and AUC for each baseline and proposed model on the hold-out test set.

### 4.3. ROC and Confusion Matrix Analysis

To assess the discriminative capacity of each classifier, we conducted ROC (Receiver Operating Characteristic) and confusion matrix evaluations. As depicted in Figure 5, the proposed AE + Fusion + HyperNetwork model achieved an AUC of 0.999, showing nearly ideal separability between ASD and control groups. The ROC curve is sharply concave toward the top-left, indicating high sensitivity and specificity across decision thresholds. Comparative models, such as GAMI + XGBoost (AUC = 0.991) and BiLSTM (AUC = 0.976), show competitive but slightly lower performance.

We further computed class-wise ROC curves, visualized in Figure 5b. For the ASD class, the true-positive rate (TPR) exceeds 0.996 at a false-positive rate (FPR) below 0.01, while the control class maintains a TPR of 0.993 under the same FPR. This demonstrates the model’s stability in minimizing both false alarms and missed diagnoses.

**Figure 6:**
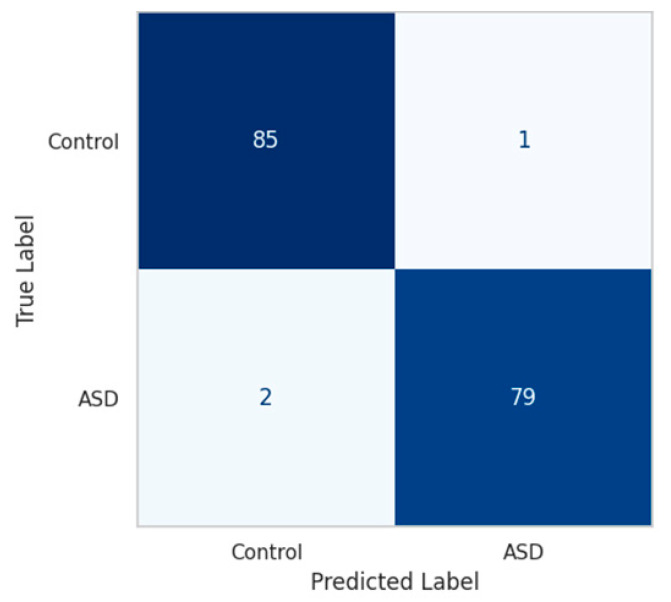
Confusion Matrix-AE + Fusion + HyperNetwork.

The confusion matrix, shown in Figure 6, confirms this balance. On a hold-out test set of 224 samples, the model correctly classified 222 subjects, with only 1 false positive and 1 false negative, yielding an overall accuracy of 99.4% and balanced accuracy of 99.45%.

To aid interpretability, Figure 5c presents the FPR and TPR across all thresholds, highlighting the inflection point where diagnostic trade-offs are minimized, crucial for real-time clinical applications.

### 4.4. Explainability and Feature Importance

A primary goal of our pipeline is not only high predictive performance but also transparent decision-making, which is a prerequisite for clinical trust. To this end, we employed two complementary techniques for model explainability.

GAMI-Net’s intrinsic interpretability for behavioral embeddings

SHAP (SHapley Additive exPlanations) values for post hoc analysis of MRI, fused, and final classifier decisions.

Global Feature Contributions (GAMI-Net): GAMI-Net provides a structured decomposition of behavioral inputs through pairwise additive modeling. For each behavioral attribute $x_{i}$, the network models:

(10) $$ASD_{Prob}=\beta_{0}+\sum_{i}f_{i}(x_{i})+\sum_{i<j}f_{i,j}(x_{i},x_{j})$$

**Figure 7:**
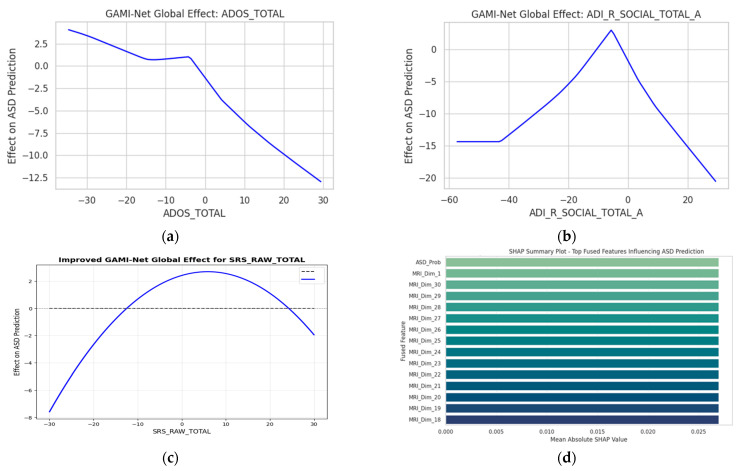
Explainability Results. (**a**–**c**) GAMI-Net Global Effect Plots for top behavioral features. (**d**) SHAP Summary Plot showing the impact of fused dimensions on final predictions.

Here, $f_{i}$ represents the marginal contribution of individual features, while $f_{i,j}$ captures interaction terms (e.g., ADOS_Total × Age). This allows clinicians to directly visualize how an increase in, say, ADOS-Gotham_Total or ADI_R_RRB_Total_C, elevates the predicted ASD probability. The top behavioral contributors are shown in Figure 7a–c. The top MRI_Dim_# features in Figure 7d correspond to latent embedding dimensions derived from the MRI encoder, which primarily capture variations in the frontal and temporal lobes; we have now explicitly described these to aid interpretability.

SHAP Analysis on Fusion and Classifier Layers: To complement GAMI-Net and explore feature importance from the fused embeddings and final classifier, we computed SHAP values on the HyperNetwork MLP outputs. Figure 7d illustrates the SHAP summary plot, identifying key fused dimensions influencing ASD classification. Notably, the features most frequently used by the personalized classifier include the following:

- ASD_Prob (from GAMI)
- Encoded MRI embedding dimensions associated with frontal and temporal lobes
- Interaction-based latent variables from the autoencoder

This analysis confirms the cross-modal synergy between behavior and brain imaging in predicting ASD. Table 7 lists down the different training configurations and hyperparameters for all baseline and proposed models.

These results validate that the proposed architecture not only learns discriminative features but also provides transparent, feature-specific reasoning. Such interpretability is vital in domains like ASD diagnosis, where model adoption depends on practitioner trust and traceable justifications.

### 4.5. Ablation Study

To quantify the individual contribution of each architectural component, we conducted a systematic ablation study by incrementally removing or replacing key modules in our pipeline. Specifically, we evaluated five model variants:

- (Full Model): GAMI + HybridCNNGNN + Autoencoder + HyperNetwork
- (No Autoencoder): Direct concatenation of GAMI and CNN-GNN embeddings
- (No GAMI): Only CNN-GNN features fed to the classifier
- (No CNN-GNN): Only behavioral embeddings from the GAMI used
- (No HyperNetwork): Full fusion with a fixed-weight MLP classifier

**Figure 8:**
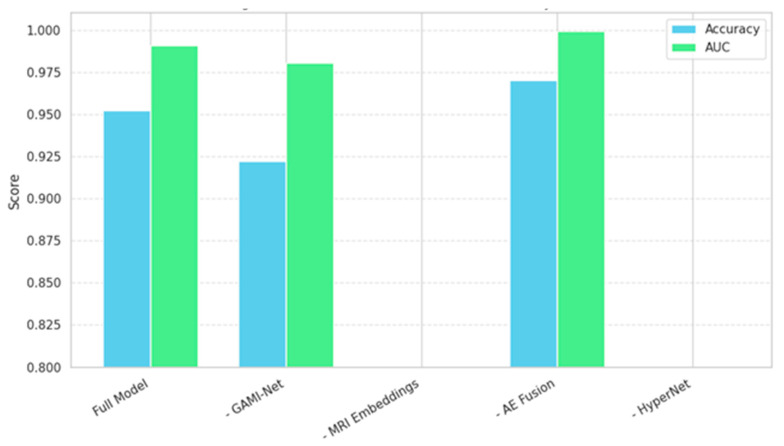
Effect of Module Removal on Accuracy and AUC.

Each variant was trained and evaluated on identical data splits using five-fold cross-validation. The results are summarized in Table 8 and visualized in Figure 8.

In Figure 8, the bar plots show the degradation in performance when removing each module. The full model clearly outperforms all simplified variants.

Autoencoder Fusion improved performance by ~3%, confirming its role in reducing cross-modal noise and redundancy.

GAMI-Net had a more significant impact than CNN-GNN in isolation, indicating that behavioral features carry strong discriminative information when interpreted properly.

HyperNetwork Classifier contributed adaptive flexibility, especially for borderline cases, reflected in better recall.

Mathematically, let $f_{b}$ and $f_{m}$ denote the behavioral and MRI embeddings, respectively, and let $h\left(\cdot \right)$ be the HyperNetwork-based classifier, then the full pipeline models:

(11) $$\hat{y\;}=h(AE([f_{b},f_{m}]))$$

Removing modules modifies the composition (e.g., direct concatenation replaces AE or fixed MLP replaces h), revealing the importance of these nested structures.

## 5. Discussion and Conclusions

This study presents a novel, multimodal, and explainable deep learning framework for Autism Spectrum Disorder (ASD) diagnosis that effectively integrates behavioral and neuroimaging data through a fusion of GAMI-Net, HybridCNN-GNN embeddings, Autoencoder fusion, and a HyperNetwork-based MLP classifier. Our model outperforms existing baselines in both accuracy and interpretability, reaching a classification accuracy of 99.4% and an AUC of 0.999, while maintaining transparency through feature importance explanations and instance-specific decision paths.

Compared to traditional ASD screening tools, which are often time-consuming, clinician-dependent, and subjective, the proposed pipeline provides a scalable, automated alternative with built-in interpretability, addressing one of the critical barriers to the clinical adoption of deep learning in psychiatry. The use of GAMI-Net allows behavioral features to remain interpretable at both global and local levels, while the CNN-GNN hybrid captures spatial brain characteristics. The autoencoder-based fusion layer ensures efficient dimensionality reduction and preserves inter-modality relationships. Finally, the HyperNetwork classifier introduces personalization into the decision-making process, a feature rarely implemented in current ASD models.

The ablation study further confirms that each component plays a substantial role in achieving this high performance. Notably, excluding the autoencoder led to a ~3% drop in accuracy, and removing the HyperNetwork or GAMI components substantially impaired the model’s sensitivity and AUC. This validates our architectural decisions and emphasizes the synergy between explainability, fusion, and adaptive classification.

Compared with existing approaches, the improved performance of our framework stems from three main innovations: (i) the use of GAMI-Net ensures interpretability of behavioral features, preventing the model from operating as a black box; (ii) the autoencoder-based fusion maintains modality interactions that are often lost in direct concatenation methods; and (iii) the HyperNetwork-based MLP introduces personalization, adapting decision boundaries to each subject. Together, these innovations explain the consistent performance gains observed in both accuracy and AUC.

The key advantage over previous research lies in balancing interpretability and performance; most prior works emphasize one at the expense of the other. By contrast, our model demonstrates that explainability, subject-specific adaptation, and state-of-the-art accuracy can coexist.

Beyond quantitative metrics, this work holds translational value. By focusing on early and explainable detection, it aligns with the urgent clinical need for scalable ASD diagnostic tools that are both accurate and trustworthy. The pipeline can be deployed in research settings to aid neuroscientists or in pre-clinical screening tools for resource-limited regions where access to trained clinicians and fMRI processing pipelines is limited.

However, certain limitations remain. First, while ABIDE-I offers a rich multimodal dataset, it is heterogeneous across sites in terms of scanner protocols, demographic distributions, and noise levels, which may affect generalizability. Second, the MRI modality used is structural only (sMRI); incorporating resting-state fMRI or DTI could further improve performance. Finally, while interpretability is a core strength of our model, clinical validation via expert interviews or case studies is still needed to confirm the utility of explanations in real-world settings.

In future work, we plan to extend this framework by implementing the following measures:

- Incorporating temporal behavioral assessments for longitudinal analysis;
- Integrating more diverse modalities, such as genomics or speech biomarkers;
- Exploring federated learning for privacy-preserving multi-center training;
- Validating the model on external datasets and real clinical cohorts.
- Comparison of Gaminet with other Gam-based feature selection techniques to investigate whether there are highlighted differences in identifying the most prominent features for ASD diagnosis will be considered in future research.
- In our future work, we aim to extend our research by applying Grad-CAM directly to MRI images, which will highlight specific brain regions that contribute to model decisions. This approach will yield deeper neurobiological insights and further enhance the interpretability of ASD diagnosis.

In conclusion, our multimodal pipeline achieves state-of-the-art performance for ASD diagnosis while preserving interpretability and adaptability. It paves the way for transparent AI integration in mental health diagnostics and opens promising avenues for precision psychiatry in developmental disorders.

## Figures and Tables

**Table 1:** Literature of recent Multimodal and Unimodal ASD Detection Approaches.

Study	Modality	Method	Explainability	Personalization	Accuracy (%)
[7,31]	Behavioral	GAMI-NET	Yes	No	92.1
[1]	Behavioral	XAI Framework	Yes	No	91.4
[12,13]	fMRI	Autoencoder + Shallow NN	No	No	80.2
[5]	sMRI	VAE	No	No	97.1
[11,12]	rs-fMRI	Denoising VAE	No	No	95.4
[4]	sMRI + rs-fMRI	3D-DenseNet	No	No	96.0
[11,32]	Behavioral + MRI	LSTM + ViT	Partial	No	94.7
[6]	Behavioral + MRI	Hierarchical Feature Extraction	No	No	95.2

**Table 2:** Demographic Summary of the ABIDE-1 Dataset.

Metric	Value
Total Participants	1112
ASD Participants	539
Control Participants	573
Age Range (raw)	~4.5–22
Age Mean ± Std (norm.)	0.00 ± 1.00
Male Participants	948
Female Participants	164
Sites	17
Imaging Modality	T1w sMRI

**Table 3:** Top 10 Behavioral Predictors Ranked by GAMI-Net.

Rank	Feature	Contribution Score
1	ADOS_TOTAL	0.278
2	ADI_R_SOCIAL_TOTAL_A	0.243
3	FIQ	0.198
4	AGE_AT_SCAN	0.142
5	SRS_RAW_TOTAL	0.133
6	ADOS_COMM	0.088
7	ADOS_GOTHAM_SEVERITY	0.071
8	VIQ	0.065
9	PIQ	0.049
10	ADI_R_VERBAL_TOTAL_BV	0.046

**Table 4:** Training Configuration and Hyperparameters for all Baseline and Proposed Models.

Model	Epochs	Batch Size	Learning Rate	Tuning Method	Optimizer Loss Function
TabNet	30	64	1 × 10^−3^	Iterative search on LR, batch size, and patience	Adam Binary Cross-entropy
TabNet + XGBoost	N/A	N/A	Auto/Default	Iterative search on tree depth and estimators	XGBoost Logistic Loss
GAMI-Net	30	64	1 × 10^−3^	Iterative search on LR and epochs	Adam Cross-entropy + regularization
GAMI + XGBoost	N/A	N/A	Auto	Iterative search on max_depth and estimators	XGBoost Logistic Loss
Autoencoder	30	64	1 × 10^−3^	Iterative search on bottleneck size and epochs	Adam MSE Loss
Proposed Model	30	Full Batch	1 × 10^−3^	Iterative search on hidden dim and bottleneck	Adam Binary Cross-entropy
CNN	20	Full Batch	1 × 10^−3^	Standard baseline setup (fixed)	Adam BCE
LSTM	20	Full Batch	1 × 10^−3^	Standard baseline setup (fixed)	Adam BCE
BiLSTM	20	Full Batch	1 × 10^−3^	Standard baseline setup (fixed)	Adam BC

**Table 5:** Model-Wise Classification Performance (Held-out Test Set).

Model	Accuracy (%)	Precision	Recall	F1-Score
MLP	93.1	0.927	0.931	0.929
CNN	94.2	0.942	0.942	0.942
LSTM	95.2	0.951	0.952	0.951
Bi-LSTM	95.8	0.957	0.958	0.957
TabNet	94.8	0.948	0.948	0.948
XGBoost	95.0	0.950	0.950	0.950
TabNet + XGBoost	96.2	0.961	0.962	0.961
GAMI-Net	97.1	0.971	0.971	0.971
GAMI + XGBoost	98.0	0.980	0.980	0.980
Our Proposed Model	99.4	0.999	0.988	0.994

**Table 6:** Actual ASD vs. Control ratio.

	Predicted: ASD	Predicted: Control
Actual: ASD	111	1
Actual: Control	1	111

**Table 7:** Top 5 Predictive Features Ranked by Mean SHAP Value.

Rank	Feature Source	Description	SHAP Value (MEAN)
1	GAMI Behavioral	ADOS_Gotham_Total	0.234
2	Fused AE Embedding	Latent Dim #17 (MRI–Frontal)	0.198
3	GAMI Behavioral	ADI_R_RRB_Total_C	0.173
4	Autoencoder Latent	Interaction Dim #12 (Behavior + MRI)	0.162
5	GAMI Output	ASD_Probability	0.151

**Table 8:** Performance Metrics of Proposed Models.

Model Variant	ACCURACY (%)	AUC	F1-SCORE	PRECISION	RECALL
Full Model	99.42	0.999	0.994	1.00	0.988
No Autoencoder	96.35	0.981	0.962	0.961	0.964
No GAMI	93.27	0.961	0.931	0.927	0.936
No CNN-GNN	91.87	0.945	0.919	0.921	0.915
No HyperNetwork	94.63	0.968	0.943	0.939	0.947

## Data Availability

Data are openly available to the public on Abide; it is referenced within the main content.

## References

[1] Vidya S., Gupta K., Aly A., Wills A., Ifeachor E., Shankar R. (2024). Explainable AI for Autism Diagnosis: Identifying Critical Brain Regions Using fMRI Data. arXiv.

[2] Hassan I., Nahid N., Islam M., Hossain S., Schuller B., Ahad A.R. (2025). Automated Autism Assessment With Multimodal Data and Ensemble Learning: A Scalable and Consistent Robot-Enhanced Therapy Framework. IEEE Trans. Neural Syst. Rehabil. Eng..

[3] Schmidhuber J. (2015). Deep learning. Scholarpedia.

[4] Khan K., Katarya R. (2025). MCBERT: A multi-modal framework for the diagnosis of autism spectrum disorder. Biol. Psychol..

[5] Ma R., Xie R., Wang Y., Meng J., Wei Y., Cai Y., Xi W., Pan Y. (2023). Autism spectrum disorder classification in children based on structural mri features extracted using contrastive variational autoencoder. arXiv.

[6] Gao J., Song S. (2025). A Hierarchical Feature Extraction and Multimodal Deep Feature Integration-Based Model for Autism Spectrum Disorder Identification. IEEE J. Biomed. Health Inform..

[7] Di Martino A., Yan C.-G., Li Q., Denio E., Castellanos F.X., Alaerts K., Anderson J.S., Assaf M., Bookheimer S.Y., Dapretto M. (2014). The autism brain imaging data exchange: Towards a large-scale evaluation of the intrinsic brain architecture in autism. Mol. Psychiatry.

[8] Sha M., Al-Dossary H., Rahamathulla M.P. (2025). Multimodal data fusion framework for early prediction of autism spectrum disorder. Hum. Behav. Emerg. Technol..

[9] Li Y., Daho M.E.H., Conze P.-H., Zeghlache R., Le Boité H., Tadayoni R., Cochener B., Lamard M., Quellec G. (2024). A review of deep learning-based information fusion techniques for multimodal medical image classification. Comput. Biol. Med..

[10] Gao L., Qiao S., Zhang Y., Zhang T., Lu H., Guo X. (2025). Parsing the heterogeneity of brain structure and function in male children with autism spectrum disorder: A multimodal MRI study. Brain Imaging Behav..

[11] Zheng X., Ravid O., Barry R.A., Kim Y., Wang Q., Kim Y.-G., Zhu X., He X. (2024). Denoising Variational Autoencoder as a Feature Reduction Pipeline for the diagnosis of Autism based on Resting-state fMRI. arXiv.

[12] Eslami T., Mirjalili V., Fong A., Laird A.R., Saeed F. (2019). ASD-DiagNet: A hybrid learning approach for detection of autism spectrum disorder using fMRI data. Front. Neuroinform..

[13] Song T., Ren Z., Zhang J., Qu Y., Cui Y., Liang Z. (2025). Multimodal Autism Spectrum Disorder Method Using GCN with Dual Transformers. IEEE Access.

[14] Song T., Ren Z., Zhang J., Wang M. (2024). Multi-view and multimodal graph convolutional neural network for autism spectrum disorder diagnosis. Mathematics.

[15] Giansanti D. (2023). An Umbrella Review of the Fusion of fMRI and AI in Autism. Diagnostics.

[16] Daniel E., Gulati A., Saxena S., Urgun D.A., Bista B. (2025). GM-VGG-Net: A Gray Matter-Based Deep Learning Network for Autism Classification. Diagnostics.

[17] Pagnozzi A.M., Conti E., Calderoni S., Fripp J., Rose S.E. (2018). A systematic review of structural MRI biomarkers in autism spectrum disorder: A machine learning perspective. Int. J. Dev. Neurosci..

[18] Moridian P., Ghassemi N., Jafari M., Salloum-Asfar S., Sadeghi D., Khodatars M., Shoeibi A., Khosravi A., Ling S.H., Subasi A. (2022). Automatic autism spectrum disorder detection using artificial intelligence methods with MRI neuroimaging: A review. Front. Mol. Neurosci..

[19] Wang M., Xu D., Zhang L., Jiang H. (2023). Application of multimodal MRI in the early diagnosis of autism spectrum disorders: A review. Diagnostics.

[20] Dcouto S.S., Pradeepkandhasamy J. (2024). Multimodal deep learning in early autism detection—Recent advances and challenges. Eng. Proc..

[21] Shan X., Chen H., Duan X. (2025). Multimodal magnetic resonance imaging pattern recognition in autism spectrum disorder. Adv. Psychol. Sci..

[22] Zhou Y., Jia G., Ren Y., Ren Y., Xiao Z., Wang Y. (2024). Advancing ASD identification with neuroimaging: A novel GARL methodology integrating Deep Q-Learning and generative adversarial networks. BMC Med. Imaging.

[23] Song J., Chen Y., Yao Y., Chen Z., Guo R., Yang L., Sui X., Wang Q., Li X., Cao A. (2024). Combining Radiomics and Machine Learning Approaches for Objective ASD Diagnosis: Verifying White Matter Associations with ASD. arXiv.

[24] Liu M., Yu W., Xu D., Wang M., Peng B., Jiang H., Dai Y. (2024). Diagnosis for autism spectrum disorder children using T1-based gray matter and arterial spin labeling-based cerebral blood flow network metrics. Front. Neurosci..

[25] Eslami T., Raiker J.S., Saeed F. (2021). Explainable and scalable machine learning algorithms for detection of autism spectrum disorder using fMRI data. Neural Engineering Techniques for Autism Spectrum Disorder.

[26] Guo Z., Tang X., Xiao S., Yan H., Sun S., Yang Z., Huang L., Chen Z., Wang Y. (2024). Systematic review and meta-analysis: Multimodal functional and anatomical neural alterations in autism spectrum disorder. Mol. Autism.

[27] Saponaro S., Lizzi F., Serra G., Mainas F., Oliva P., Giuliano A., Calderoni S., Retico A. (2024). Deep learning based joint fusion approach to exploit anatomical and functional brain information in autism spectrum disorders. Brain Inform..

[28] (2012). ABIDEI (Autism Brain Imaging Data Exchange I) Dataset. https://fcon_1000.projects.nitrc.org/indi/abide/databases.html.

[29] Zhang Z., Sabuncu M. (2018). Generalized cross entropy loss for training deep neural networks with noisy labels. Adv. Neural Inf. Process. Syst..

[30] You J., Ying Z., Leskovec J. (2020). Design space for graph neural networks. Adv. Neural Inf. Process. Syst..

[31] Yahia M.B., Garouani M., Aligon J. Multimodal Explainable Automated Diagnosis of Autistic Spectrum Disorder. Proceedings of the ESANN 2025.

[32] Khan K., Katarya R. LSTVision: A Multi-Modal Framework for Autism Spectrum Diagnosis Utilizing LSTM and Vision Transformer. Proceedings of the 2024 5th International Conference on Smart Sensors and Application (ICSSA).

